# The epidemiology of traumatic brain injuries in the fastest-paced city in China: a retrospective study

**DOI:** 10.3389/fneur.2023.1255117

**Published:** 2023-11-09

**Authors:** Jun-feng Zou, Hai-lan Fang, Jing Zheng, Yu-qiang Ma, Chu-wei Wu, Gao-jian Su, Xian-sheng Liu, Jun Liu, Jie Gao, Jie-hua Zhang, Dong-liang Zhu, Xin Shi, Xian-jian Huang

**Affiliations:** ^1^Department of Neurosurgery, Shenzhen Key Laboratory of Neurosurgery, The First Affiliated Hospital of Shenzhen University, Shenzhen Second People’s Hospital, Shenzhen, China; ^2^Department of Emergency, Shenzhen Hyzen Hospital, Shenzhen, China; ^3^Healthcare Services Department, Yantai City Health Comprehensive Service Center, Yantai, China; ^4^Shenzhen Health Development Research and Data Management Center, Shenzhen Municipal Health Commission, Shenzhen, China; ^5^Department of Neurosurgery, Peking University Shenzhen Hospital, Shenzhen, China; ^6^China Medical University, Shenyang, China; ^7^School of Maths and Information Science, Shandong Technology and Business University, Yantai, China

**Keywords:** traumatic brain injury, epidemiology, public health, cross-sectional study, China

## Abstract

**Introduction:**

Traumatic brain injury (TBI) seriously affects the quality of human health and the prognosis of the patient, but the epidemiological characteristics of TBI can vary among populations. Numerous changes have occurred in the epidemiological characteristics of individuals with TBI in the fast-paced city of Shenzhen, China. However, little is known about these characteristics. This study aimed to investigate the changes in TBI epidemiology, help clinicians improve medical treatment.

**Methods:**

In this retrospective cross-sectional analysis, we collected the data of 4,229 patients with TBI admitted to 20 hospitals in Shenzhen in 2017. We collected data on age, gender, cause and severity of the injury, eventual diagnosis, time from injury to admission in a neurosurgery department, and patient outcomes. Two neurosurgeons simultaneously collected the data. We compared these results with a similar study conducted in Shenzhen during the period from 1994 to 2003 to clarify and explain the changes in the epidemiological characteristics of TBI.

**Results:**

The majority of respondents were men [2,830 (66.9%)]. The mean age was 32.5 ± 21.4 years. The youngest patient was less than 1 year old, and the oldest patient was 101 years old. A total of 3,947 (93.3%) patients had a favorable outcome, 219 (5.2%) had an unfavorable outcome, and 63 (1.5%) died. The predominant external cause was falls (1,779 [42.1%]); this was the most common cause of TBI in children and older adults. Riders of electric bicycles (423 [29.0%]) were the most vulnerable to traffic accident-related injuries. Time greater than 50 h from injury to admission to a neurosurgical department had a significant effect on prognosis (*p* < 0.001).

**Conclusion:**

The epidemiological characteristics of TBI have changed significantly over the past 20 years. Falls, rather than traffic accidents, were the most common cause of TBI. Further research is needed to devise solutions to decrease the incidence of falls and improve the outcomes of TBI.

## Introduction

Traumatic brain injury (TBI) seriously affects human health worldwide, especially severe TBI, which is associated with poor prognosis and heavy trouble for families and society (1).

The 2019 Global Burden of Disease study has estimated that of the 4 million deaths from external injuries worldwide, nearly 90% are in low- and middle-income countries (2). With the development of industrialization and demographic changes, the major burden of disease in low- and middle-income countries has shifted from infectious diseases and nutritional diseases to non-communicable diseases and trauma. The burden of trauma in low-income countries is expected to increase in the coming years (3).

The TBI treatment system and the level of treatment vary greatly among regions and countries. Improving the connection between different TBI treatment systems and the continuity of the entire treatment process should be the priority of social health agendas (3).

Since China’s reform and opening up 40 years ago, the economy has developed rapidly. In the rapid urban development process, the variety of jobs and activities, especially the variety of manual labor jobs and outdoor activities, has significantly increased, resulting in a substantial increase in the incidence of TBI (4, 5). Over the past 30 years, Chinese neurosurgeons have focused their attention on the epidemiology of TBI. Several provincial and regional epidemiological studies have been published, but national data on the incidence of TBI in China are not available (4). Several large-scale Chinese studies conducted in the 1980s indicated that the incidence of TBI was 55.4–64 cases per 100,000 people annually (6–8). Accordingly, there are approximately 772,800–892,800 new cases of TBI per year in China (4, 6–8). In 2016, another global study indicated that the incidence in China was 313 per 100,000 people annually, while the prevalence was 742 per 100,000 people (9). In developed countries, for example, the United States, 823.7 cases of TBI occur per 100,000 people annually (1).

Shenzhen, one of China’s special economic zones and the city with the fastest pace has shown rapid development (10). It is the first fully urbanized city in China, with no rural areas or agricultural land in recent decades (11). It is representative of China’s rapid urban development. Shenzhen is divided into main urban areas and surrounding urban areas due to different government management policies (12).

In 1979, Shenzhen’s population was only 314,000 (13); the city has rapidly expanded into a modern metropolis with a population of more than 10 million people in recent decades. Several young migrant adults moved to Shenzhen and had children; thus, young domestic immigrant families formed the majority of the city population. Young men have been employed in many heavy industries in Shenzhen, resulting in a much higher incidence of TBI in men than in women.

In 2017, there were 12.53 million permanent residents in Shenzhen (13). Marked changes have taken place for industrial safety, communication, and transportation. These changes have greatly affected the characteristics and incidence of TBI (4). On the contrary, with the migration of the aging population to all Chinese cities, the causes of TBI have changed significantly (4). To effectively reduce the incidence of TBI, policymakers and clinicians need to understand the epidemiological characteristics and changing trends of TBI in Shenzhen.

The causes of TBI vary greatly between countries, cities, and even stages of urban development (1, 3, 4). A thorough understanding of the changes in TBI epidemiology can help physicians understand the epidemiological characteristics of TBI and policymakers adopt corresponding preventive measures to reduce the occurrence of TBI. Thus, this study aimed to investigate the changes in TBI epidemiology and help clinicians and policymakers improve medical treatment and formulate appropriate social regulations.

## Methods

This study was conducted by the Neurosurgery Department of the First Affiliated Hospital of Shenzhen University (Shenzhen Second People’s Hospital), a key national department. This study received approval from the government and concerned hospitals; informed consent was waived, and the study design was approved by the ethics review board of the First Affiliated Hospital of Shenzhen University (Shenzhen Second People’s Hospital) (KS2019062806). Data were obtained from previous medical records. It was not appropriate or possible to involve patients or the public in the design, conduct, reporting, or dissemination plans of this research.

In 2017, 40 hospitals were qualified to treat patients with TBI and perform TBI surgery in Shenzhen. We selected 20 of these hospitals through random sampling and collected information about patients with TBI. We designed a survey registration form to collect data on admitted TBI patients between 1 January 2017 and 31 December 2017. Data were collected from all inpatients with TBI, including those transferred to the neurosurgery department during hospitalization. The following characteristics were analyzed within 30 days after admission: age, gender, cause, and severity of the injury, diagnosis, and patient outcomes. We also collected data on the time from injury to admission in a neurosurgery department, including the duration of transport of patients to the hospital and observation in the emergency department. Diagnoses and injury causes were coded using the International Classification of Diseases, 10th Revision. We classified the causes into five categories: falls (unintentional), traffic accidents, violence, exposure to other external forces (e.g., being struck accidentally by a person or object), and unknown or unspecified causes. TBI severity was defined using standard definitions based on the Glasgow Coma Scale (GCS) (14). The Glasgow Outcome Scale (GOS) and GCS were recorded by the attending physician at admission and discharge, respectively. If the GCS changed within 3 days after the injury, the GCS on the third day was used for assessing the severity. If the patient performed surgical treatment during this period, we evaluated the severity using the pre-surgical GCS. The outcome was considered favorable if the GOS was five or four and unfavorable if it was three or two, and if the patient died, the GOS was scored as one (4). The prognosis was assessed by GOS at the end of treatment (discharge) or on the 30th day if the patient was hospitalized at that time. Data collection was performed by two neurosurgeons simultaneously.

Between 1994 and 2003, Huang et al. conducted an epidemiological survey involving 10,607 patients with TBI in all inpatient departments of 35 hospitals in Shenzhen (15). The results of our study were compared with those of Huang et al.’s survey. Eight age groups were established: children and teens (0–9 years and 10–19 years), young adults (20–29 years and 30–39 years), middle-aged (40–49 years and 50–59 years), and older adults (60–69 years and 70 years or older) for easy comparison of the age distribution of the patients.

The age was represented by mean +/− standard deviation (SD), and a comparison of the ages of men and women was performed using a *t*-test. Pearson’s chi-square test was used to compare the proportion of gender, age group, accident causes, and differences in results between the two surveys. When the assumption for the chi-square test was violated, Fisher’s exact test was used. The prognostic factors of TBI were analyzed by binary logistic regression. The data were analyzed using IBM SPSS Statistics (version 26).

## Results

A total of 4,229 patients admitted for TBI were identified, with 2,829 men (66.9%) and 1,400 women (33.1%). The mean age of the TBI inpatients was 32 years. The youngest patient was less than 1 year old, and the oldest patient was 101 years old. There were no significant differences in age between men and women in TBI (Table 1; *p* = 0.28).

**Table 1:** Mean age in TBI inpatients.

Gender	*N* (%)	Age (Mean ± SD^a^)	*t*	*p*-value
Male subjects	2,830 (66.9%)	32.73 ± 20.49	1.043	0.297
Female subjects	1,399 (33.1%)	32.00 ± 23.23		
Total	4,229	32.49 ± 21.43		

^a^SD, standard deviation.

**Figure 1:**
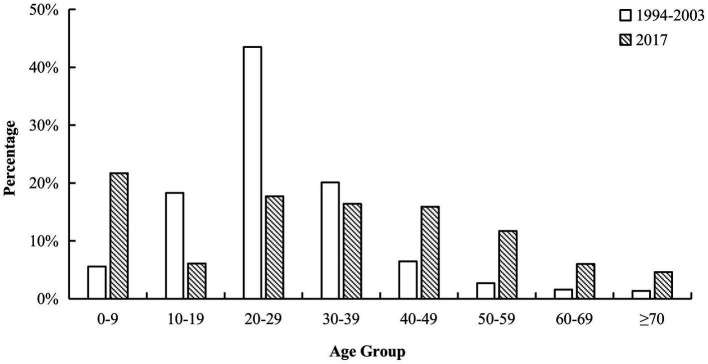
Age specific of TBI inpatients. The bar chart contains eight age groups, 0 to 9 years old, 10 to 19 years old, 20 to 29 years old, 30 to 39 years old, 40 to 49 years old, 50 to 59 years old, 60 to 69 years old, and 70 years old and older. The white columns represent the percentage of corresponding age groups in the previous study and the black columns represent the results of our study. The age distributions in the two studies were significantly different (*p* < 0.001). There are 0.5% inpatient with unclear age in the previous survey. TBI, traumatic brain injury.

Young adults were the largest part of this study (*n* = 1,443 [34.1%]). The proportion of children and teens (*n* = 1,174 [27.8%]) was close to that of middle-aged people (*n* = 1,165 [27.5%]; Figure 1).

**Figure 2:**
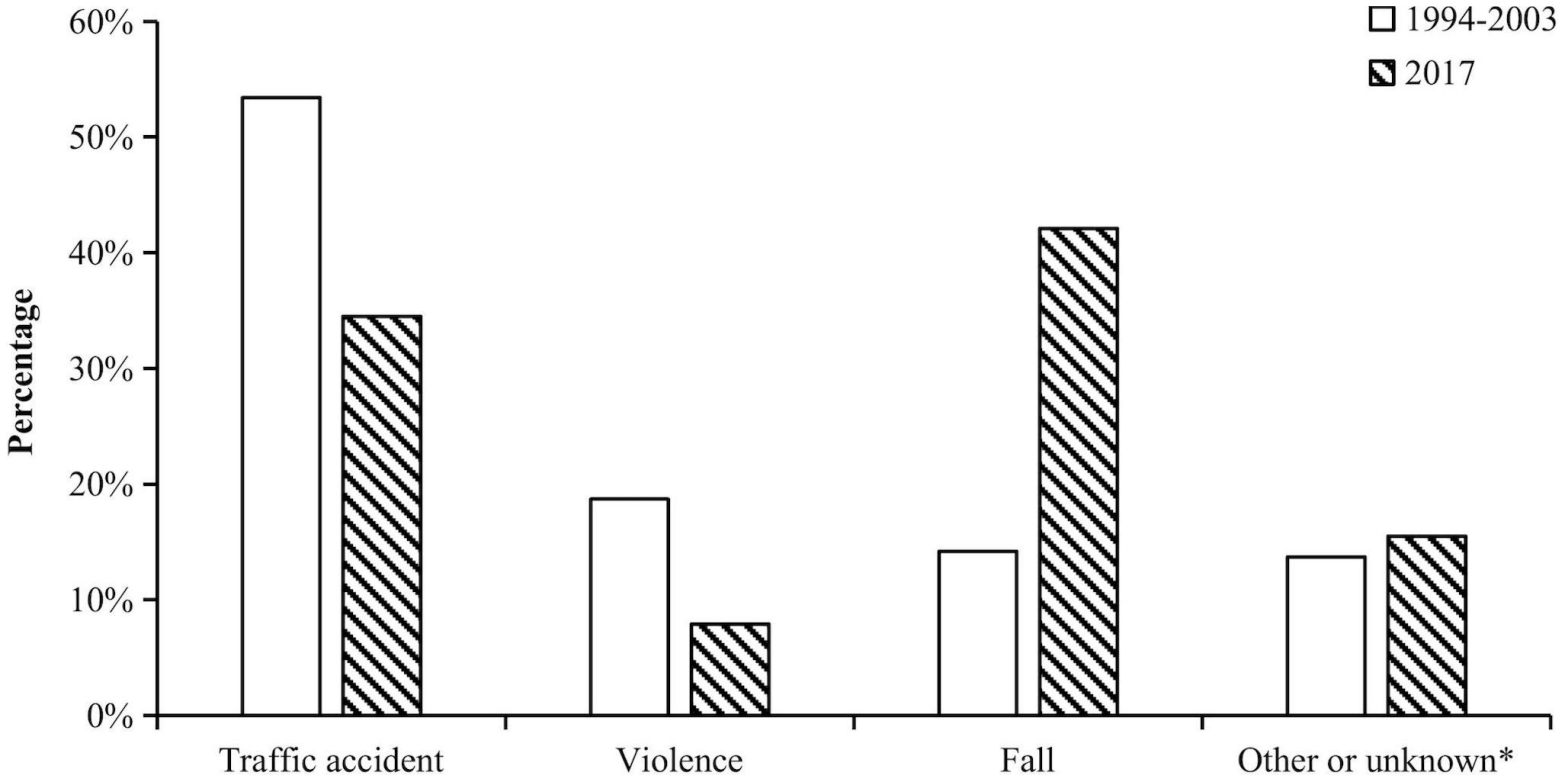
Causes of TBI. Injury causes were divided into four categories in previous study. For comparison, external force injuries in this study were merged to other or unknown groups. Other or unknown group included the cause of exposure to external forces, because the previous study did not have this cause group. The white columns represent the percentage of corresponding groups in the previous study and the black columns represent the results of our study. The causes of TBI in the two studies were significantly different (*p* < 0.001).

Fall injuries (*n* = 1,779 [42.1%]) were the most common cause of TBI, followed by injuries related to transportation accidents (*n* = 1,459 [34.5%]). There were 332 (7.9%) patients with TBI with violent injuries (Figure 2).

**Figure 3:**
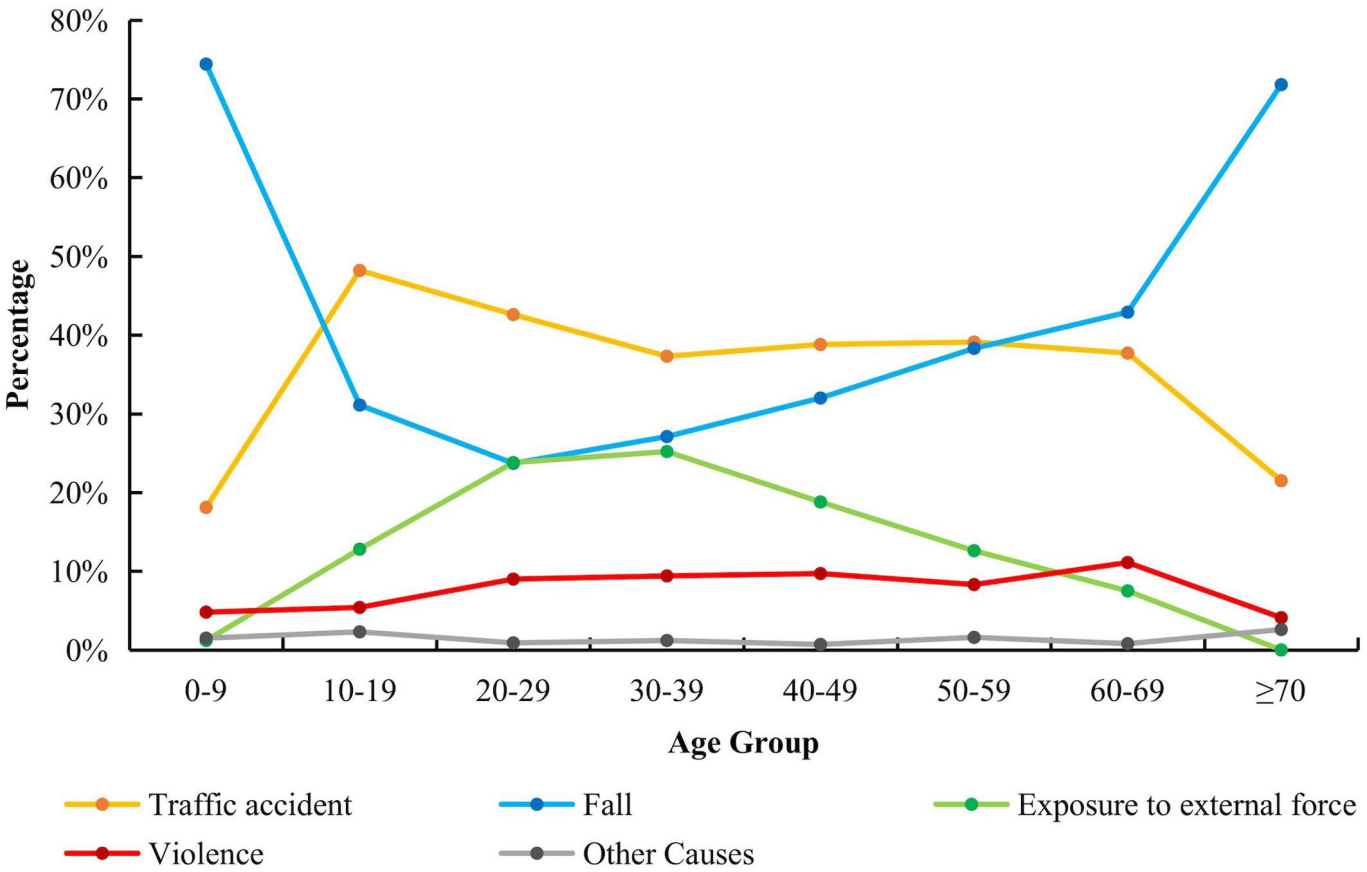
Causes of TBI in all age group. This line chart shows the proportion of five causes of TBI in all age groups. The causes of TBI in each age group were significantly different (*p* < 0.001).

The 20- to 29-year-old age group (23.7%) had the lowest incidence of fall-related injuries. Conversely, the youngest (74.4%) and oldest (71.8%) age groups presented the highest incidence of fall-related injuries (Figure 3). The proportion of injuries related to traffic accidents in those aged 10–69 years was high. However, this was much lower in children under 10 years of age and in older people over 70 years of age.

**Figure 4:**
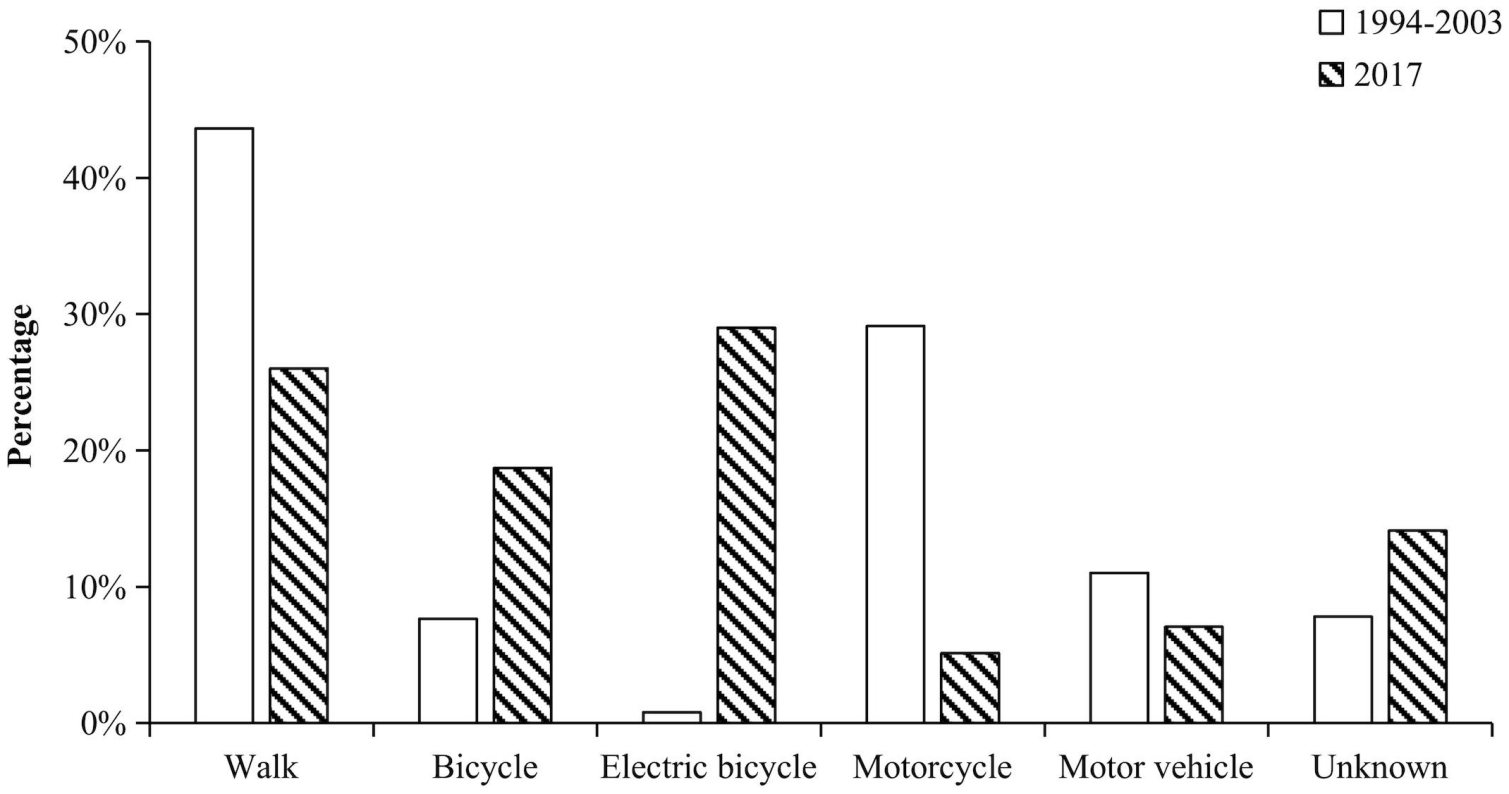
Transportation used in traffic accidents. The bar chart contains six groups, walk, bicycle, electric bicycle, motorcycle, motor vehicle and other transportation. The white columns represent the percentage of corresponding groups in the previous study and the black columns represent the results of our study. The transportation used in traffic accidents in the two studies were significantly different (*p* < 0.001).

Among injuries related to traffic accidents, injuries due to electric bicycles were the most common (*n* = 423 [29.0%]), followed by pedestrian injuries (*n* = 379 [26.0%]) and bicycle-related injuries (*n* = 273 [18.7%]; Figure 4).

According to the severity distribution in this study, mild TBI occurred in 3,731 patients (88.2%), moderate TBI in 177 patients (4.2%), and severe TBI in 238 patients (5.6%). Severity was not assessed in 83 patients (2.0%) due to the patient’s inebriated state or other reasons. Traffic accident-related injuries (*n* = 134 [56.3%]) were the most common cause of severe TBI, followed by fall-related injuries (*n* = 69 [29.0%]).

Head CTs were performed on all enrolled patients before admission. Skull fracture occurred in 1,713 patients (40.5%), skull base fracture in 695 patients (16.4%), brain contusion in 1,306 patients (30.9%), acute epidural hematoma in 586 patients (13.9%), acute subdural hematoma (ASDH) in 842 patients (19.9%), traumatic subarachnoid hemorrhage in 1,114 patients (26.3%), traumatic hemorrhage in the brain tissue in 254 patients (6.0%), and traumatic intraventricular hemorrhage in 34 patients (0.8%). Surgical treatment was performed on 588 patients (13.9%), of whom 430 (73.1%) had a favorable outcome. In total, 330 patients (7.8%) performed hematoma evacuation, 153 patients (3.6%) performed unilateral decompressive craniectomy, and 26 patients (0.6%) performed bilateral decompressive craniectomy.

**Figure 5:**
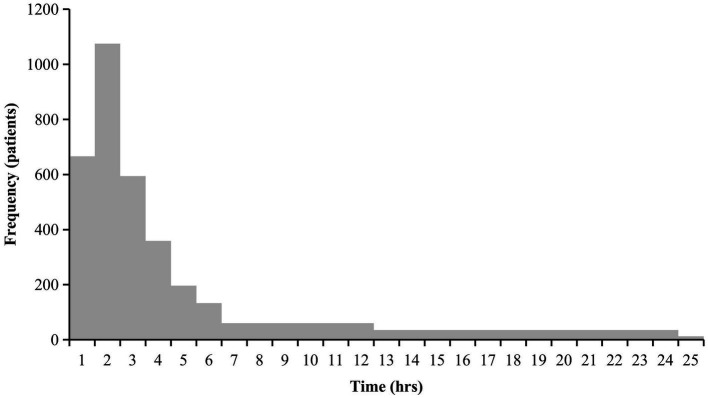
The time from injury to presenting at a department of neurosurgery. This histogram shows the time from injury to presenting at a department of neurosurgery. The time from injury to presenting at a department of neurosurgery was divided into 1 hours, 2 hours, 3 hours, 4 hours, 5 hours, 6 hours, 7–12 hours, 12–24 hours, 25–50 hours and over 50 hours. The portion exceeding 25h is not shown because of fewer number.

Unfavorable outcome predictors for TBI inpatients, such as age, severity, pupillary light reflex, Babinski sign, types of injuries (closed or open), ASDH, hemorrhage in brain tissue, and the time from injury to admission in a neurosurgery department, significantly affected patient outcomes (Table 2). When the time from injury to admission in a neurosurgery department was longer than 50 h, the prognosis of the patients was significantly worse (*p* < 0.001; Figure 5).

**Table 2:** Unfavorable outcome predictors for patients with TBI.

Variates	OR	95%CI	*p*-value
Age	1.027	1.020–1.035	<0.001
Severity			
Mild	Ref		
Moderate	4.706	2.923–7.577	<0.001
Severe	9.412	5.726–15.469	<0.001
Pupillary light reflex			
Normal	Ref		
Both absent	2.229	1.441–3.449	<0.001
Babinski sign			
Negative	Ref		
Positive	2.243	1.236–4.070	0.008
Types of injuries			
Closed	Ref		
Open	1.491	1.073–2.072	0.017
ASDH			
Without	Ref		
With	2.557	1.854–3.525	<0.001
Hemorrhage in the brain tissue			
Without	Ref		
With	2.053	1.352–3.118	0.001
Time from injury to admission in a department of neurosurgery			
>50 h	Ref		
<50 h	0.528	0.320–0.869	0.012

ASDH, acute subdural hematoma. Multi-variable logistic regression model for risk factors (odds ratio with 95% confidence interval) showed that unfavorable outcome and death (GOS 1-3) predictors for patients with TBI included increasing age, severity, pupillary light reflex, Babinski sign, types of injuries, ASDH, hemorrhage in the brain tissue, time from injury to admission in a department of neurosurgery.

In total, 3,947 patients (93.3%) had a favorable outcome, 219 patients (5.2%) had an unfavorable outcome, and 63 patients (1.5%) died during hospitalization. The outcomes of patients with different severities of brain injury were significantly different (*p* < 0.001; Table 3), with unfavorable outcomes, and death, in 2.6 and 53.4% of patients with mild and severe TBI, respectively.

**Table 3:** Outcomes of TBI inpatients.

Severity	Favorable outcome (%)	Unfavorable outcome (%)	Death (%)	Total (%)
Mild	3,634 (97.4%)	80 (2.1%)	17 (0.5%)	3,731 (88.2%)
Moderate	136 (76.8%)	38 (21.5%)	3 (1.7%)	177 (4.2%)
Severe	111 (46.6%)	86 (36.1%)	41 (17.2%)	238 (5.6%)
Unknown	66 (79.5%)	15 (18.1%)	2 (2.4%)	83 (2.0%)
Total	3,947 (93.3%)	219 (5.2%)	63 (1.5%)	4,229

This table shows the number and proportion of outcome indifferent severity of traumatic brain injury, and they were significantly different.

## Discussion

The epidemiological characteristics of TBI, such as age, injury causes, and prognoses, have changed significantly since previous findings. Huang et al.’s research was conducted in the early stages of China’s reformation and opening up, while our research was conducted nearly 40 years later. Therefore, the results of the two studies evaluated the epidemiological characteristics of TBI at different levels of economic development.

The results of the research by Huang et al. indicated that the majority of TBI patients were between 20 and 29 years old, and the ratio of men to women with TBI was 2.79:1. However, the urban population of Shenzhen had a male/female ratio of 1:7; thus, the actual ratio of men to women with TBI was estimated at approximately 20:1 at that time (15). Our results indicated that the majority of patients were young adults (mean age = 34 years), but the proportion of patients who were middle-aged and older adults has increased significantly.

The early city dwellers migrated to Shenzhen decades ago; their descendants constituted the young adult group in this study. Subsequently, Shenzhen became an attractive city for young adults to settle down in. Therefore, most inpatients with TBI continue to be young adults in Shenzhen. Meanwhile, social aging is increasing in Shenzhen, similar to other cities in China. The proportion of middle-aged and older patients with TBI has also increased (16).

The previous study indicated that the number of male patients with TBI was significantly higher than that of female patients, reflecting the gender composition of the local population 20 years ago. Most of the city dwellers were men; therefore, the proportion of men with TBI in Shenzhen was significantly higher than that in developed countries. In 2017, the male/female ratio of the Shenzhen population structure was approximately 1:1, while the male/female ratio of inpatients with TBI was approximately 2:1, which has not changed significantly according to data from 20 years ago. However, more detailed research is needed to determine the reason for this.

The causes of injury reflect the urban development and changes in Shenzhen. In the previous study, Shenzhen was in a period of rapid urban development, with a large increase in the number of vehicles used. Traffic accident-related injuries were the most common cause of TBI, followed by violent injuries and falls. Excluding walking, the most common means of transportation for patients with TBI were motorcycles, followed by cars, but only rarely were electric bicycles involved. Similarly, between 1990 and 2016, many epidemiological studies of TBI conducted in China indicated that traffic accident-related injuries were the most common cause, accounting for approximately 50% of cases (4). Furthermore, in Huang et al.’s study, violence-related injuries were more common than fall-related injuries probably because the population in Shenzhen was young (15).

The occurrence of TBI is related to the economic state and the traffic management system of the city. In 2003, the Shenzhen government banned motorcycles in the city (17). Thus, motorcycle-related TBIs accounted for a very small percentage of traffic accident-related injuries (5.1%) in this study, constituting a significant decrease from that in the previous study. In April 2012, the use of electric bicycles was restricted in the city (17). Despite this, the rapid increase in electric bicycles has led to alarming amounts of electric bicycle-related TBIs, similar to the rates of motorcycle-related TBIs in the past.

Regarding traffic accident-related injuries, the 20- to 29-year-old age group used electric bicycles the most. A large proportion of this age group included migrants from other cities with low income, which required cheap and convenient commuting. At the same time, the takeout industry and the express delivery industry, aimed at providing food and daily necessities to customers on time, have been developed in Shenzhen. As the number of deliveries they complete dictates their income, they attempt to deliver the maximum amount of goods as quickly as possible.

Electric bicycles are often necessary for people to live and work. They can help people commute between bus stations, subway stations, and destinations, which is cheaper and more convenient than taking a taxi or driving a car. However, the management of electric bicycles is still relatively difficult. It has both the flexibility of bicycles and a relatively fast speed. Electric bicycle riders often arbitrarily switch on the sidewalks and car lanes; hence, pedestrians and car drivers have no time to avoid them. We propose that all electric bicycles need a license plate before they can be used on the road and that penalties should be toughened to limit random violations of traffic regulations.

Building bike lanes may also be a very important initiative to reduce electric bicycle-related brain injuries. People living in Beijing know that riding a bike is very convenient and safe because of the bike lanes, but there are very few bike lanes in Shenzhen. We believe that the government should pay more attention to the construction of bicycle lanes, whether it is to facilitate people’s going out, reduce the occurrence of TBI, or promote green travel. Limiting the speed of electric bicycles is a good way to reduce the number of accidents related to electric bicycles. In 2019, the Traffic Law came into force, stipulating that the speed of electric bikes should not exceed 25 km per hour (18). However, there are still many owners who ask sellers to refurbish electric bicycles, and more efforts should be made to limit related illegal activities. Building electric bicycle lanes is also a good idea. Although the construction period may be relatively long and difficult, there should be a significant reduction in accidents related to electric bicycles after completion.

Traffic accidents and accident-related injuries have been a problem since China began to reform and open up. A European study indicated that crashes in road traffic accidents were the most common cause of admission to the intensive care unit (19). Similarly, traffic accidents (135 [56.3%]) were the most common cause of severe TBI in Shenzhen, followed by falls (70 [29.2%]). These results are consistent with those of some European studies (20).

The increasing rate of falls should be considered. In contrast to some previous studies, fall injuries were the primary cause of TBI in this study, although we only recruited inpatients. However, some other studies showed that unintentional falls were the most common cause of TBI among admissions to emergency departments. Age was significantly correlated with the rate of falls (19, 21, 22). In children (0–9 years old) and older people (over 70 years old), fall injury was the main cause of TBI, and its incidence was significantly higher in these groups than in other age groups. These findings are particularly concerning, as chronic subdural hemorrhage can develop several weeks after a head injury in older people (23, 24).

In the last 4 decades, millions of young adults have migrated to Shenzhen for work, and their parents subsequently migrated to care for their children (12). Accompanied by the aging society and the two-child policy of family planning in China, the proportions of both young children and older adults in the population have increased. Therefore, the number of falls among older adults and young children has increased. Thus, we should pay more attention to older adults and young children to reduce the risk of falls. This can be achieved through the encouragement of safety education in high-risk populations and families, which can be conducted in homes and public places.

In addition to the growth of the economy, Shenzhen’s medical technology has also improved significantly compared to the past. The total hospital mortality rate decreased from 4.3 to 1.5% compared to the data for the period 1994–2003 (15).

CT has been popularized in Shenzhen hospitals, greatly improving the accuracy of diagnosis. Although traumatic hemorrhage in the brain tissue and ASDH are relatively rare, no matter the amount of bleeding, the prognosis is significantly worse. At the same time, we should also pay attention to physical examinations. Bilateral pupil light reflex is worse (both sides, dull or disappearance; one side, dull, and the other side, disappearance), and the Babinski sign (one or both sides) suggests a significantly worse prognosis. This is important for both neurosurgeons and emergency department physicians.

It should be noted that the time from injury to admission in a neurosurgery department significantly affected patient outcomes. The prognosis of the patient worsened if the time was greater than 50 h. Therefore, the duration of observation and transport in the emergency department should be less than 50 h from the time of injury. To do this, it is first necessary to improve public awareness of TBI and transfer patients with TBI to hospitals, particularly those equipped to treat TBI, as soon as possible, even when the patient’s condition does not appear serious. In addition, patients with TBI should be admitted to the neurosurgery department for further treatment. When an exact diagnosis may not be made immediately, timely referral will mitigate the deterioration of the condition due to delayed treatment.

With the development of modern society and the economy in China, people and the government are paying more attention to the development of health services. Governments can improve national awareness of safety and reduce the incidence of TBI by educating people about safety and implementing responsible safety systems. To reduce the mortality and disability rates of TBI, the government should address the gaps in diagnosis and treatment between regions and hospitals.

This study has a few limitations. We only enrolled 4,229 inpatients with TBI and excluded patients treated in emergency departments or community health departments. Furthermore, we only recorded TBI events for over a year. More detailed data are needed to draw accurate conclusions in future studies. However, the data source was limited to Shenzhen, China, and was therefore more relevant to developing countries.

## Conclusion

The epidemiological characteristics of TBI in Shenzhen have changed with social progress and gradual improvements in traffic laws and regulations. Between 1994 and 2003, traffic accident-related injury was the main cause of TBI inpatients, followed by violencerelated TBI. However, fall-related injuries were the main cause, followed by traffic accident-related injuries in 2017. Therefore, more attention should be paid to these demographics. Between 1994 and 2003, the motorcycle-related TBI was the most important type of traffic accident-related injury, while in 2017, it was the electric bicycle-related TBI. This also indirectly shows the importance of this kind of transportation in people’s lives. The management of electric bicycles was a very important part of reducing TBI, just like the management of motorcycles was in the past. The epidemiological characteristics of TBI are important aspects of urban modernization as they reflect the quality of regulation and healthcare. This epidemiological study may help physicians and policymakers understand the epidemiology of TBI and reduce the incidence in Shenzhen, thus improving the prognosis of TBI.

## Data availability statement

The raw data supporting the conclusions of this article will be made available by the authors, without undue reservation.

## Ethics statement

The studies involving human participants were reviewed and approved by the Ethics Review Board of the First Affiliated Hospital of Shenzhen University, Shenzhen Second People’s Hospital. Written informed consent from the patients/participants or patients/participants' legal guardian/next of kin was not required to participate in this study in accordance with the national legislation and the institutional requirements.

## Author contributions

JuZ: Writing – original draft. HF: Writing – original draft. JiZ: Writing – original draft. YM: Writing – original draft. CW: Writing – original draft. GS: Writing – original draft. XL: Writing – original draft. JL: Writing – original draft. JG: Writing – original draft. JieZ: Writing – original draft. DZ: Writing – original draft. XS: Writing – review & editing. XH: Writing – review & editing.

## References

[1] Maas AIR Menon DK Adelson PD Andelic N Bell MJ Belli A. Traumatic brain injury: integrated approaches to improve prevention, clinical care, and research. Lancet Neurol. (2017) 16:987–1048. doi: 10.1016/S1474-4422(17)30371-X 29122524

[2] Maas AIR Menon DK Manley GT Abrams M Akerlund C Andelic N. Traumatic brain injury: progress and challenges in prevention, clinical care, and research. Lancet Neurol. (2022) 21:1004–60. doi: 10.1016/S1474-4422(22)00309-X 36183712 PMC10427240

[3] GBD 2019 Diseases and Injuries Collaborators. Global burden of 369 diseases and injuries in 204 countries and territories, 1990-2019: a systematic analysis for the global burden of disease study 2019. Lancet. (2020) 396:1204–22. doi: 10.1016/S0140-6736(20)30925-9 33069326 PMC7567026

[4] Jiang JY Gao GY Feng JF Mao Q Chen LG Yang XF. Traumatic brain injury in China. Lancet Neurol. (2019) 18:286–95. doi: 10.1016/S1474-4422(18)30469-1 30784557

[5] Zou JF Huang XJ Wu CW Su GJ. Discussion on part of existing problems in the epidemiology of traumatic brain injury in China. Chin J Neurot Surg. (2021) 7:059–62. doi: 10.3877/cma.j.issn.2095-9141.2021.01.014

[6] Zhu GL Song JR Zhang DX Wang WZ Xu ZL. The epidemiology of head injury in rural and minority areas of China. Chin J Neurosurg. (1989) 5:44–7.

[7] Yang YC Li SC Cheng XM Wang WZ Wu SP. The epidemiology of craniocerebral injury in 6 cities of China. Chin J Neurosurg. (1987) 3:23–5.

[8] Wang CC Schoenberg BS Li SC Yang YC Cheng XM Bolis CL. Brain injury due to head trauma. Epidemiology in urban areas of the People's Republic of China. Arch Neurol. (1986) 43:570–2. 3718283 10.1001/archneur.1986.00520060034013

[9] GBD 2016 Traumatic Brain Injury and Spinal Cord Injury Collaborators. Global, regional, and national burden of traumatic brain injury and spinal cord injury, 1990–2016: a systematic analysis for the global burden of disease study 2016. Lancet Neurol. (2019) 18:56–87. doi: 10.1016/S1474-4422(18)30415-0 30497965 PMC6291456

[10] Xinhua Net. Comprehensive economic competitiveness of Chinese cities. Available at: http://www.xinhuanet.com/local/2017-06/23/c_1121198564.htm (Accessed April 21, 2019).

[11] People’s Daily. Shenzhen will become the country's first city without rural residents. Available at: http://data.people.com.cn/r2004mrb/20040630/5 (Accessed June 1, 2020).

[12] Statistics Bureau of Shenzhen Municipality. Shenzhen statistical yearbook 2019. Beijing: China Statistics Press (2019). 53–57 p.

[13] Office of Local Chronicles Compilation of Shenzhen City. Social life. Available at: http://www.sz.gov.cn/cn/zjsz/nj/content/post_1356006.html (Accessed June 1, 2020).

[14] Teasdale G Jennett B. Assessment of coma and impaired consciousness. A practical scale Lancet. (1974) 2:81–4. 4136544 10.1016/s0140-6736(74)91639-0

[15] Huang XP Chen JL Jiang HP Zhang H Liu K Zhang FL. Analysis of TBI inpatients epidemiologic characters between 1994 and 2003 in Shenzhen. Chin J Modern Med. (2007) 17:1088–91. doi: 10.3969/j.issn.1005-8982.2007.09.018

[16] Yang C Lang LJ He Z Hui JY Jiang JY Gao GY. Epidemiological characteristics of older patients with traumatic brain injury in China. J Neurotrauma. (2022) 39:850–9. doi: 10.1089/neu.2021.0275 35171687

[17] Shenzhen Special Zone Daily. A joint conference established on Banning motorcycles and restricting electricity in Shenzhen. Available at: http://www.sz.gov.cn/cn/xxgk/zfxxgj/zwdt/content/post_1493827.html (Accessed June 1, 2020).

[18] Safety technical specification for electric bicycle. National public service platform for standars information (Accessed November 20, 2022).

[19] Steyerberg EW Wiegers E Sewalt C Buki A Citerio G De Keyser V. Case-mix, care pathways, and outcomes in patients with traumatic brain injury in CENTER-TBI: a European prospective, multicentre, longitudinal, cohort study. Lancet Neurol. (2019) 18:923–34. doi: 10.1016/S1474-4422(19)30232-7 31526754

[20] Peterson AB Kegler SR. Deaths from fall-related traumatic brain injury – United States, 2008-2017. MMWR Morb Mortal Wkly Rep. (2020) 69:225–30. doi: 10.15585/mmwr.mm6909a2 PMC7367089 32134910

[21] Haarbauer-Krupa J Haileyesus T Gilchrist J Mack KA Law CS Joseph A. Fall-related traumatic brain injury in children ages 0-4years. J Saf Res. (2019) 70:127–33. doi: 10.1016/j.jsr.2019.06.003 PMC6927527 31847987

[22] Feigin VL Theadom A Barker-Collo S Starkey NJ McPherson K Kahan M. Incidence of traumatic brain injury in New Zealand: a population-based study. Lancet Neurol. (2013) 12:53–64. doi: 10.1016/S1474-4422(12)70262-4 23177532

[23] Sahyouni R Goshtasbi K Mahmoodi A Tran DK Chen JW. Chronic subdural hematoma: a historical and clinical perspective. World Neurosurg. (2017) 108:948–53. doi: 10.1016/j.wneu.2017.09.064 28935548 PMC12747318

[24] Yang WY Huang J. Chronic subdural hematoma: epidemiology and natural history. Neurosurg Clin N Am. (2017) 28:205–10. doi: 10.1016/j.nec.2016.11.002 28325454

